# The Effects of *Aspergillus fumigatus* Colonization on Lung Function in Patients with Cystic Fibrosis

**DOI:** 10.3390/jof7110944

**Published:** 2021-11-09

**Authors:** Mahasin Al Shakirchi, Kimmo Sorjonen, Lena Klingspor, Peter Bergman, Lena Hjelte, Isabelle de Monestrol

**Affiliations:** 1 Stockholm Cystic Fibrosis Centre, Karolinska University Hospital Huddinge, 141 86 Stockholm, Sweden; lena.hjelte@ki.se (L.H.); isabelle.demonestrol@ki.se (I.d.M.); 2 Department of Clinical Science, Intervention and Technology, Division of Pediatrics, Karolinska Institutet, 171 77 Stockholm, Sweden; 3 Department of Clinical Neuroscience, Division of Psychology, Karolinska Institutet, 171 77 Stockholm, Sweden; kimmo.sorjonen@ki.se; 4 Department of Laboratory Medicine, Division of Clinical Microbiology, Karolinska Institutet, 171 77 Stockholm, Sweden; lena.klingspor@ki.se (L.K.); peter.bergman@ki.se (P.B.); 5 Department of Infectious Diseases, The Immunodeficiency Unit, Karolinska University Hospital Huddinge, 141 86 Stockholm, Sweden

**Keywords:** cystic fibrosis, fungi, *Aspergillus fumigatus*, lung function

## Abstract

*Aspergillus fumigatus* is commonly isolated from CF airways. However, the impact on CF lung progression is not completely understood. In this study, using a 16-year retrospective observational cohort study (2000–2015) that included 132 patients, we determined the annual lung function, measured as percent predicted forced expiratory volume in the first second (ppFEV1), decline before and after the first colonization with *A. fumigatus*. Further, in the same individual, the ratios of lung function when patients were colonized with *A. fumigatus* and when they were not were calculated. The impact of eradication, with antifungal treatment or spontaneously, was assessed. The annual ppFEV1 was significantly lower after the first colonization with *A. fumigatus*. Furthermore, within the same individual, colonization with *A. fumigatus* for two and three years in a row was associated with 4.3% and 7.9% lower ppFEV1, respectively, compared to when not colonized. Finally, patients who eradicated *A. fumigatus* the following two years after colonization exhibited 9.9% and 14.5% higher ppFEV1 compared to patients who continued to produce cultures with *A. fumigatus* for two and three years. Our study demonstrated that *A. fumigatus* colonization was associated with a negative impact on lung function in the long term and eradication, spontaneously or with treatment, was associated with a better pulmonary outcome.

## 1. Introduction

Cystic fibrosis (CF) is the most common lethal hereditary disease in the Caucasian population. Morbidity and mortality in CF are mainly related to lung disease manifestations. The inability to clear inhaled bacteria and fungi leads to their persistence and potential infection. Unlike bacteria, the impact and management of fungi in the CF lung progression is still unclear. *Aspergillus fumigatus*, the most common mold in humans, is frequently recovered from the CF airways [1,2]. Novel experiments have demonstrated an impaired mucociliary transport and an inadequate innate and adaptive antifungal immune response resulting in reduced ability to clear *A. fumigatus* in CF [3]. Moreover, an exaggerated antifungal inflammatory response to *A. fumigatus* is reported in animal CF models [4,5,6]. In a CF murine model, *A. fumigatus* infection resulted in increased levels of interleukin (IL)-1 α, IL-1 β and IL-9, leading to an uncontrolled detrimental inflammatory response [4,6,7]. Furthermore, a dysregulation of Th17/Treg cells in response to *A. fumigatus* has been described and restoring the regulation of this immunological axis with kynurenines improved antifungal immunity [5]. An interesting study, and the first to address the antifungal immune response in CF in humans, has shown that the level of reactive oxygen species (ROS) produced by phagocytes in response to *A. fumigatus* were significantly higher than in healthy controls and that ROS-levels were correlated to CF disease severity [8].

In terms of clinical outcomes, the impact of *A. fumigatus* colonization on CF lung disease progression has been studied with so far conflicting results. Amin et al. showed that chronic colonization with *A. fumigatus* was associated with a decline in lung function and an increased risk of hospitalization due to lung exacerbations [9]. Furthermore, CF patients with recurrent exacerbations tended to be colonized with *A. fumigatus* to a larger extent [10]. Interestingly, even early acquisition of *Aspergillus* species cultured in bronchoalveolar lavage (BAL) from infants with CF was associated with a decline in lung function at early school age [11]. Furthermore, high-resolution computed tomography (HRCT) detected early abnormalities in the lungs of CF patients colonized with *A. fumigatus*, even with unaltered lung function [12,13]. Similarly, it was recently shown in children with CF that *Aspergillus* infections were associated with progression of structural lung disease and trapped airways as shown by HRCT [14]. Providing a different perspective, a cross-sectional study has shown that CF patients who were cultured positive for *A. fumigatus* had a worse respiratory quality of life [15]. Notably, there are other studies where the negative impact of *A. fumigatus* colonization on lung function could not be confirmed [12,13,16,17], possibly due to a short observational period [12], a cross sectional study design [13] or the use of different fungal detection methods [17].

Given the uncertainties in this field, the main objective of the current study was to assess the impact of *A. fumigatus* colonization on lung function. Further, the impact of *A. fumigatus* eradication, spontaneously or with antifungal treatment, on lung function was determined. Finally, parameters which may influence the impact of *A. fumigatus* colonization on lung function were studied.

## 2. Materials and Methods

### 2.1. Study Design

The current study was a 16-year retrospective observational cohort single center study (2000–2015). As there is no agreed definition of *A. fumigatus* colonization in the CF context, researchers in the field adopted various definition. We studied the impact of colonization, defined as the presence of *A. fumigatus* in sputum culture ≥1/year, ≥2/year and according to Leeds definition criteria, on lung function. Leeds criteria were developed for *P. aeruginosa* in CF patients and define chronic colonization as >50% positive sputum cultures and intermittent colonization as ≤50% positive sputum cultures in the last 12 months with a minimum of four cultures per patient and year [18]. However, we decided to focus on the presence of *A. fumigatus* in fungal culture at least once at a given year. The results for the other definitions are presented as supplements (Tables S1 and S2).

#### 2.1.1. The Impact of *A. fumigatus* Colonization on Lung Function

The impact of *A. fumigatus* colonization on lung function, measured as percent predicted forced expiratory volume in the first second (ppFEV1), was estimated in two ways:

a. The annual predicted lung function decline before and after the first colonization with *A. fumigatus*.
b. Within the same individual, we estimated the ratios of ppFEV1 when patients were colonized with *A. fumigatus* and when they were not. Since alteration in lung function could be delayed, we determined lung functions for five different time-ranges: (a) the same year of colonization, (b) one year after colonization, (c) two years after colonization, (d) colonization with *A. fumigatus* for two years in a row and (e) colonization with *A. fumigatus* for three years in a row.

#### 2.1.2. The Impact of *A. fumigatus* Eradication on Lung Function

Because of the wide variation in treatment indication, treatment duration and lack of data about antifungal serum level concentration in the study period, the efficacy of antifungal treatment could not be determined. Instead, we investigated the impact of eradication on lung function. Eradication was defined as conversion of a positive fungal culture for *A. fumigatus* in sputum or BAL to a negative one with or without antifungal treatment and remaining negative throughout the following 12 months. Based on the eradication status the following two years after colonization, four conditions were identified and the ratios of lung function were calculated between these four conditions.

(a) YNN (Y = yes, N = no) Colonization with *A. fumigatus* year one and eradication year two and three
(b) YNY Colonization with *A. fumigatus* year one and three and eradication year two
(c) YYN Colonization with *A. fumigatus* year one and two and eradication year three
(d) YYY Colonization with *A. fumigatus* al three years

#### 2.1.3. Possible Predictors of the Impact of *A. fumigatus* on Lung Function

Parameters, such as gender, age, *CFTR* genotype, 25-OH vitamin D levels, Body Mass Index (BMI) in adults, pancreas function, erythrocyte sedimentation rate (ESR), total IgG, number of intravenous antibiotic treatments, CF-related diabetes (CFRD) and co-colonization with *P. aeruginosa*, *Candida albicans* and *Candida dubliniensis*, that may contribute to either inhibition or promotion of the predicted lung function decline associated with *A. fumigatus* colonization in CF patients were assessed.

### 2.2. Study Population

CF patients with confirmed CF diagnosis who solely attending Stockholm CF center 2000–2015 and were above the age of 5 years were included. CF diagnosis was based on CF clinical symptoms and two positive sweat tests or two CF causing mutations [19]. Patients who underwent lung transplantation were excluded after the lung transplant. Lung function tests were performed during the annual assessments at the division of Clinical Physiology, Karolinska University Hospital Huddinge. Data were retrieved from the Swedish CF registry and medical record.

### 2.3. Fungal Cultures

Sputum and BAL fluid were treated with Sputolysin (1:1) before further handling. Aspergillus species were identified by macro- and microscopy according to the laboratory standard protocol of the microbiology laboratory at Karolinska University Hospital, Huddinge. Specimens were cultured on Sabouraud dextrose agar with antibiotics (gentamicin and/or chloramphenicol) at 30 °C for 7 days and potato dextrose agar at 42 °C for 7 days before being moved to 30 °C for an additional 3 days.

### 2.4. Statistical Methods

The effects in the present study, e.g., of the age × before/after first positive fungal culture for *A. fumigatus* interaction effect on the natural logarithm of ppFEV1, were calculated with linear mixed models, and the effects were allowed to vary between participants, i.e., they were defined as random. This means that the effects were calculated within, rather than between, the participants. Analyses were conducted with R 4.0.2 statistical software [20] employing the lmerTest package [21]. We used the natural logarithm of ppFEV1, rather than raw ppFEV1, as the outcome variable, as we believe that the same absolute change in ppFEV1 should be viewed differently depending on the initial value. For example, we believe that a decrease of 10 percent units in ppFEV1 is more severe if it is from 20 to 10 than from 100 to 90. An additional advantage of using a logarithmic scale is that you would never predict a negative ppFEV1, which may happen if using raw ppFEV1. As for the interpretation of coefficients, if the effect of age, in years, on the natural logarithm of ppFEV1 equals, for example, −0.02, this would mean that the ratio of ppFEV1 at a specific year and the year before, i.e., ppFEV1_year_/ppFEV1_year−1_ or ppFEV1_year+1_/ppFEV1_year_, would be predicted to equal e^−0.02^ = 0.98 and this ratio would mean that ppFEV1 is predicted to decrease by 2% (1 − 0.98 = 0.02) per year.

Further, the impact of colonization on lung function was measured by calculating the ratio ppFEV1_colonization/_ppFEV1_non colonization_ within the same individual. A ratio <1 means that ppFFV1 was worse when the patient was colonized and vice versa. For example, a ratio of 0.95 would indicates 5% (1 − 0.95 = 0.05) lower lung function when the patient was colonized compared to when not colonized.

Similarly, the effect of eradication was calculated by measuring the ratio ppFEV1_for a specific condition_/ppFEV1_for the compared condition_. A ratio of 1.145 indicate 14.5% (1.145 − 1 = 0.145) higher lung function in a specific condition than in the compared condition.

Finally, the odds ratio (OR) was calculated to assess which parameters can affect the outcome of colonization with *A. fumigatus* on lung function and in what way. An OR > 1 would indicate a protection and OR < 1 would predict a promotion of the potential negative effect of colonization with *A. fumigatus*.

## 3. Results

### 3.1. Patients Characteristics

The clinical characteristics of the study population are presented in Table 1. In total, 132 patients were included. The number of patient-years was 1484. The mean follow-up period was 10.2 years. During the study period, 3080 fungal cultures (3074 sputum and 6 BAL) were obtained. The mean annual number of obtained fungal cultures per patient was 2.1, which increased over the last five years of the study period to 3.4. The study cohort was characterized into two groups based on the presence or absence of *A. fumigatus* in sputum or BAL cultures. The *A. fumigatus* colonizer group (*n* = 77, 58% of the study cohort) included patients who presented with *A. fumigatus* in sputum or BAL at any time in the study period. In contrast, the non-colonizer group (*n* = 55, 42% of the study cohort) included solely CF patients who were never cultured positive for *A. fumigatus* during the study period. In general, both groups shared several key features, such as gender distribution, mean age and mean lung function measured as ppFEV1. However, there were significant differences between the groups (Table 1). Pancreatic insufficiency, allergic bronchopulmonary aspergillosis (ABPA) diagnosed according to minimal ABPA criteria [22], chronic and intermittent colonization with *P. aeruginosa* and the presence of *C. albicans* were more common in the colonizer group compared to the non-colonizer group.

### 3.2. The Impact of A. fumigatus Colonization on Lung Function

#### 3.2.1. The Annual Lung Function Decline before and after the First Colonization with *A. fumigatus*

**Figure 1:**
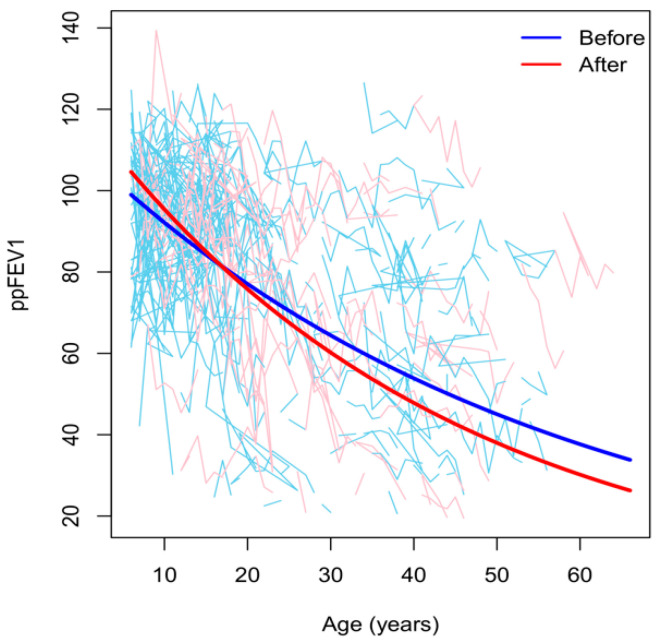
The individual observed (thin lines) and predicted (thick line) lung function trajectories before (blue) and after (red) the first colonization with *A. fumigatus*.

Lung function measured as ppFEV1 was predicted to decrease by 1.8% per year before and 2.3% per year after the first positive culture for *A. fumigatus* (*p* = 0.003), (Figure 1). However, this difference could be due to a significant acceleration in the deterioration with age, rather than a true interaction effect. Notably, after adjustment for this quadratic effect of age, the difference in the annual lung function decline before and after the first positive culture for *A. fumigatus* remained significant (*p* = 0.015). Further statistical analyses with respect to gender using three-way interaction showed that the effect of *A. fumigatus* did not vary according to gender (*p* = 0.816). The results remained similar even after excluding patient-years when patients had ABPA and/or received antifungal treatments, which theoretically could affect lung function (*p* = 0.005).

#### 3.2.2. The Impact of *A. fumigatus* Colonization on Lung Function over Five Different Time Ranges

The impact of *A. fumigatus* colonization (*n* = 77) was assessed at five time-ranges: (a) the year of detection, (b) one and (c) two years after detection and (d) if colonization persisted for two or (e) three years in a row. The ratios of ppFEV1 were calculated within the same individual and adjusted for the quadratic effect of age. The results indicated that *A. fumigatus* colonization for two (*n* = 37) or three years (*n* = 23) in a row was associated with a significant decrease in ppFEV1 by 4.3% (1 − 0.957 = 0.043 = 4.3%) and 7.9% (1 − 0.921 = 0.079 = 7.9%), respectively (*p* < 0.01), compared to ppFEV1 in non-colonization conditions within the same individual. No significant changes in lung function were observed the year of colonization or after one or two years, Table 2.

### 3.3. The Impact of A. fumigatus Eradication on Lung Function

Based on the eradication status one and two years after colonization, four different conditions were defined (described in Section 2.1.2). CF patients who cultured positive for *A. fumigatus* and converted to negative the following two years (YNN, number (*n*) of cases = 44) exhibited 9.9% (1.099 − 1 = 0.099 = 9.9%) and 14.5% (1.145 − 1 = 0.145 = 14.5%) higher ppFEV1 compared to CF patients who continued culturing *A. fumigatus* for two (YYN, *n* = 30) and three years (YYY, *n* = 59), respectively, (Table 3). Similarly, CF patients who were cultured positive for *A. fumigatus* and converted to negative the next year but re-cultured positive the third year (YNY, *n* = 16) exhibited 14.3% (1.143 − 1 = 0.143 = 14.3) higher ppFEV1 compared to CF patients who continued culturing positive for *A. fumigatus* for three years in a row (YYY) (Table 3).

However, only six cases (14%) in the group YNN received antifungal treatment and in the vast majority (*n* of cases = 38, 86%) the eradication occurred spontaneously. Further, in the group YYN, 17 cases (57%) received antifungal treatment whereas the rest eradicated *A. fumigatus* spontaneously the third year. Nevertheless, in the group YYY, 36 cases (62%) received antifungal treatment; only 12 (20%) eradicated *A. fumigatus* and 24 (41%) continued to recover *A. fumigatus* despite treatment the fourth year. Three cases (5%) in this group managed to eradicate *A. fumigatus* spontaneously the fourth year. The customary antifungal agent used in the study period was posaconazole as liquid formulation (*n* = 35). The other antifungal agents were posaconazole as tablet formulation (*n* = 9), voriconazole (*n* = 2), itraconazole (*n* = 2) and nebulized Amphotericin B (n = 1). Overall, CF patients treated with antifungal agents had 6.1% lower lung function than patients who were colonized with *A. fumigatus* and who did not receive treatment (*p* < 0.05). No significant improvement in lung function was reported one and two years after the initiation of antifungal treatment (data not shown).

### 3.4. Possible Parameters That may Predict the Impact of A. fumigatus on Lung Function

Theoretically, *A. fumigatus* colonization may affect CF patients differently. The odds ratio (OR) of the impact of the previously mentioned parameters (Section 2.1.3) that may be associated with a protection (OR > 1) or promotion (OR < 1) of the potentially negative association of *A. fumigatus* colonization in four different time ranges were assessed. Few parameters seemed to moderate the effect of *A. fumigatus* on lung function. F508del heterozygosity and co-colonization with *P. aeruginosa* showed to be associated with a protective effect against the negative association of *A. fumigatus* colonization on lung function (OR > 1), Table 4.

## 4. Discussion

Despite the great attention to *A. fumigatus* in CF lung disease, the clinical implication of this mold has not been established yet. We aimed to study the impact of *A. fumigatus* colonization and eradication on lung function. In general, our results supported the notion that *A. fumigatus* colonization is associated with lower lung function. The cardinal findings can be summarized in three main implications. First, the annual predicted lung function decline in CF accelerated after the first acquisition of *A. fumigatus*, supporting previous studies [11,23]. Second, colonization with *A. fumigatus* for more than one year in a row was associated with a significant decline in lung function. Third and most importantly, *A. fumigatus* eradication, with or without treatment, was associated with a better lung function compared to patients who continued to culture *A. fumigatus* for more than one year.

These findings raise an intriguing question: should *A. fumigatus* be treated at the very first year of detection? With the exception for ABPA, treatment with antifungal agents in *A. fumigatus* colonization is not recommended [24]. In fact, studies dealing with the efficacy of antifungal treatment of *A. fumigatus* colonization in the CF context are very scarce. The vast majority of published studies are case reports with a wide variation in treatment indications, chosen antifungal agents and treatment duration [25,26,27,28]. The only randomized placebo-controlled study on antifungal treatment in CF patients colonized with *A. fumigatus* was with itraconazole for a period of 24 weeks [23]. The study did not show a beneficial outcome in terms of lung function improvement [23]. However, the limitation of this study was that therapeutic plasma levels of itraconazole were not achieved in 43% of treated patients as well as the short follow up period [29]. Thus, the existing evidence is insufficient for making a treatment decision. Therefore, clinicians face the challenging choice “treat or leave” [30]. On one hand, the clinician could wait with the treatment as the chance to obtain a spontaneous eradication was high in the group YNN (colonization year one and eradication the following two years) and given the potential risk of side effects and the development of azole resistance [31,32,33]. On the other hand, giving no treatment could implicate a risk of a longer colonization-period with *A. fumigatus* (>1 year) which was associated with a considerable risk of lung function deterioration according to the present study. Furthermore, in the group YYY (colonization three years in a row), in whom the lung function decline was most pronounced (−14.5%), the chance to eradicate *A. fumigatus* either spontaneously or with antifungal treatment was dramatically reduced. This may indicate that it was too late to treat. Alternatively, the treatment was suboptimal, as the most common antifungal treatment used in our study was posaconazole, a solution known to have a limited oral bioavailability. In addition, in the treated patients there was a wide variation in treatment duration, follow up period, isolation rate, compliance and opportunity to measure the serum concentrations levels of antifungal agents.

Because of the nature of the retrospective design, causality cannot be determined. The association between lung function decline and *A. fumigatus* colonization for more than one year could probably be interpreted in three different ways. First, patients with advanced lung disease are to a greater extent more susceptible to persistent colonization with *A. fumigatus*. In the present study, pancreatic insufficiency and colonization with *P. aeruginosa*, markers for severe CF disease, were more prevalent in colonizers compared to non-colonizers (Table 1). This is in accord with a previous study which reported that patients who were colonized with *A. fumigatus* had lower lung function at baseline and four years before the colonization [23]. Second, it could be argued that *A. fumigatus* and the antifungal immune response are inherently deleterious to CF airways [3,4,6]. A third possible interpretation is that *A. fumigatus* may affect CF patients differently. McLean et al. investigated this hypothesis and found no variation of the mucosal and the inflammatory responses induced by different *A. fumigatus* clusters in different CF primary epithelial cells [34]. In the current study, clinical characteristics, serological or microbiological parameters as risk or protectable factors for lung function deterioration in CF patients colonized with *A. fumigatus* were assessed. Unexpectedly, the negative association of *A. fumigatus* colonization on lung function was not shown in patients co-colonized with *P. aeruginosa*. In vitro, it has been reported that *P. aeruginosa* inhibits *A. fumigatus* biofilm [35,36]. In contrast, Smith et al. showed that a cytotoxic substance, elastase, produced by *P. aeruginosa* was increased in the presence of *A. fumigatus* suggesting additive impairment in CF lungs [37]. In clinical terms, Düesburg et al. showed that co-colonization with *P. aeruginosa* and *A fumigatus* was not associated with further decline in lung function, but in patients solely colonized with *A. fumigatus* a significant decline in lung function was reported. The study of Düesburg et al. and our study share the similarity that colonization with *A. fumigatus* was not associated with impaired lung function in patients colonized with *P. aeruginosa* [38]. Notably, an Irish study reported a significant decline in lung function in CF patients co-colonized with these two pathogens [39] However, the comparison in this study was between colonizers having both *A. fumigatus* and *P. aeruginosa* and non-colonizers who were negative for both microbes [39]. To conclude, the current study could not determine whether colonization with *A. fumigatus* is a direct mediator of CF lung impairment or if *A. fumigatus* in cultures is a result of poor lung function.

The present study has a number of strengths. The main one is the high number of patient-years and the total number of fungal cultures. Moreover, the impact of *A. fumigatus* colonization on lung function has been estimated in different ways, the predicted annual lung function decline, the long- and short-term decline. Furthermore, for the first time, as we are aware of, the impact of *A. fumigatus* eradication was assessed.

One limitation of the study is that eradication was only assessed with fungal cultures which are not as sensitive as molecular methods [40,41]. Furthermore, despite the high number of fungal cultures, the mean annual number of fungal cultures per patient were relatively low, especially in non-colonizers.

Introducing *CFTR*-modulators during the last few years has been a breakthrough in CF care. However, the chronic inflammation and infection with bacteria and fungi in the CF airways are still outstanding challenges [42]. Moreover, the impact of *CFTR*-modulators on fungi has not been clarified yet. Our study highlighted some aspects regarding the role of *A. fumigatus* in the CF context. Colonization with *A. fumigatus* was associated with a lower lung function and patients who eradicated this fungus exhibited a higher lung function. Therefore, we suggest repeated fungal cultures in CF patients and to consider antifungal treatment if *A. fumigatus* persists as the probability of spontaneous eradication decreases dramatically the longer *A. fumigatus* persists. Further studies with a prospective and interventional design could add more clarity to the field with focus on the treatment aspect (when, how and whom to treat). A structured definition of *A. fumigatus* colonization in CF and a standardized mycological examination involving molecular methods are essential to perform these studies.

## Figures and Tables

**Table 1:** Patient characteristics.

	All Patients	*A. fumigatus* Colonizer	Non-Colonizer	*p*-Value
Patients, *n*	132	77	55	
Patient-year, *n*	1484	948	536	
Number of fungal culture (mean per year)	3080 (2.1)	2265 (2.4)	815 (1.5)	
Demographic characteristics				
Mean age (range), y	24.7 (6–66)	24.5 (6–64)	25.0 (6–66)	0.560 ^b^
Female/male, *n*	65/67	40/37	25/30	0.462 ^a^
Clinical features:				
*CFTR* genotype				0.089 ^a^
F508del homozygotes, *n* (%)	66 (50%)	44 (57%)	22 (40%)	
F508del heterozygotes, *n* (%)	42 (32%)	19 (25%)	23 (42%)	
Other/other, *n* (%)	24 (18%)	14 (18%)	10 (18%)	
Mean ppFEV1 (sd)	78.7 (24.9)	79.6 (24.3)	77.2 (25.1)	0.908 ^b^
Mean BMI adults (sd)	21.7 (2.5)	22.26 (2.7)	20.2 (3.9)	0.204 ^b^
Comorbidities:				
Exocrine pancreatic insufficiency, *n* (%)	117 (89%)	75 (97%)	42 (76%)	<0.001 ^a^
CFRD ^1^, *n* (%)	29 (22%)	21 (27%)	8 (15%)	0.082 ^a^
Patients with ABPA, *n* (%)	11 (8%)	10 (13%)	1 (2%)	0.022 ^a^
Treatments:				
Mean number of iv antibiotic treatments	2.7	2.8	2.6	0.061 ^b^
Number of patients treated with antifungal agents	34	33	1 ^2^	<0.001 ^a^
Microbiology:				
Patients with *P. aeruginosa* ^3^, *n* (%)	112 (89%)	73 (95%)	39 (71%)	<0.001 ^a^
Number of patient-year with *P. aeruginosa*	896	544	337	
Patient with *S. aureus*, *n* (%)	103 (78%)	63 (82%)	40 (72%)	0.214 ^a^
Number of patient-year with *S. aureus*	576	418	154	
Patients with NTM, *n* (%)	23 (18%)	17 (22%)	6 (11%)	0.095 ^a^
Number of patient-year with NTM	79	68	10	
Patients with *C. albicans*, *n* (%)	113 (86%)	71 (92%)	42 (76%)	0.011 ^a^
Number of patient-year with *C. albicans*	707	467	224	
Patients with *C. dubliniensis*, *n* (%)	37 (28%)	23 (30%)	14 (25%)	0.578 ^a^
Number of patient-year with *C. dubliniensis*	152	94	55	

^a^ Tested with a Chi-Squared Test, ^b^ Tested with Linear Mixed Model. ^1^ Both diet and insulin treated CF related Diabetes Mellitus, ^2^ Patient was treated with antifungal agents because of ABPA, ^3^ Both chronic and intermittent colonization. ABPA Allergic Bronchopulmonary Aspergillosis, IV Intravenous, NTM Nontuberculous Mycobacteria.

**Table 2:** The impact of *A. fumigatus* colonization on lung function over five different time ranges.

	The Year of Detection	One Year after Detection	Two Years after Detection	Two Years in a Row	Three Years in a Row
Colonization with *A. fumigatus*	0.988 (0.965; 1.011)	0.968 (0.937; 1.000) ^†^	0.997 (0.970; 1.024)	0.957 (0.919; 0.996) *	0.921 (0.869;0.977) **

^†^ *p* < 0.10, * *p* < 0.05, ** *p* < 0.01. Effects (ratios, with 95% CI) of colonization with *A. fumigatus* defined as ≥ 1 positive fungal culture at a given year on ppFEV1, separately for five different time ranges. The effects are calculated within individuals and adjusted for the quadratic association with age. Ratio < 1 indicates that lung function was lower when colonized and vice versa.

**Table 3:** Ratio of lung function (ppFEV1) for each pairwise comparison between four different conditions of *A. fumigatus*.

Reference	YYY (*n* = 59)	YNY (*n* = 16)	YYN (*n* = 30)	YNN (*n* = 44)
YYY	-	1.143 *	1.041	1.145 **
YNY	-	-	0.911	1.001
YYN	-	-	-	1.099 *

* *p* < 0.05, ** *p* < 0.01. YNN = Colonization with *A. fumigatus* year one and eradication year two and three. YNY = Colonization with *A. fumigatus* year one and three and eradication year two. YYN = Colonization with *A. fumigatus* year one and two and eradication year three. YYY = Colonization with *A. fumigatus* al three years. Ratio >1 indicates that lung function was higher in the conditions in the column (numerator) than the compared condition in the row (denominator).

**Table 4:** The odds ratio of parameters that may predict the impact of *A. fumigatus* on lung function.

Parameters	The Year of Detection	One Year after Detection	Two Years after Detection	Two Years in a Row
Age	0.999	0.998	1.001	1.001
Gender	1.043	0.956	1.08	1.05
Genotype 1 ^a^	0.938	1.182 ^†^	1.05	1.092
Genotype 2 ^b^	0.988	1.19 *	0.964	1.034
Pancreas insufficiency	1.01	1.011	0.97	1.093
Diabetes 1 ^c^	1.026	0.998	1.009	1.006
Diabetes 2 ^d^	NA	NA	NA	1.59
Number of iv ^e^ antibiotic treatments	0.967	0.951	0.96	0.994
25 hydroxy vitamin D	1.001	1.001	NA	1.001
BMI (adults)	1.051	0.948	0.928	1.038
ESR ^f^	0.998	1.003	0.999	1.002
IgG	0.999	0.987	0.995	1.002
Co-colonization with *C. dubliniensis*	0.874	0.913	1.012	1.002
Co-colonization with *C. albicans*	1.04	0.996	NA	1.02
Co-colonization with *P. aeruginosa*	1.115 **	1.140 **	1.108 ^†^	1.129 *

^†^ *p* < 0.10, * *p* < 0.05, ** *p* < 0.01. ^a^ F508del homozygotes, ^b^ F508del heterozygotes, ^c^ Insulin treated diabetes mellitus, ^d^ Diet treated diabetes mellitus, ^e^ intravenous, ^f^ erythrocyte sedimentation rate.

## Data Availability

The data presented in this study are available on request from the corresponding author.

## Supplementary Materials

The following are available online at https://www.mdpi.com/article/10.3390/jof7110944/s1, Table S1: The impact of *A. fumigatus* colonization adopting four different definition criteria on lung function over five different time ranges, Table S2: Possible parameters that may predict the impact of *A. fumigatus* on lung function adopting different definition.
