# Supplementary material for: The Effects of Aspergillus fumigatus Colonization on Lung Function in Patients with Cystic Fibrosis
Source: J Fungi (Basel). 2021 Nov 9;7(11):944. doi: 10.3390/jof7110944 (PMC8618016; doi:10.3390/jof7110944)
Supplement: Supplementary file 1 [file jof-07-00944-s001.zip › jof-1420435-supplementary.pdf]

Supplements:

Table S1: The impact of *A. fumigatus* colonization adopting four different definition criteria on lung function over five different time ranges.

Predictor	The year of detection	One year after detection	Two years after detection	Two years in a row	Three years in a row
***A. fumigatus* ≥1 at a given year (n=77)**	0.988 (0.965; 1.011)	0.968 (0.937; 1.000)[†]	0.997 (0.970; 1.024)	**0.957 (0.919; 0.996)***	**0.921 (0.869; 0.977)****
***A. fumigatus* ≥2 at a given year (n=43)**	0.984 (0.953; 1.017)	0.970 (0.933; 1.009)	0.979 (0.937; 1.023)	NA [a]	NA [a]
**Leeds, Intermittent (n=60)**	**0.955 (0.918; 0.992)***	0.991 (0.948; 1.035)	1.008 (0.958; 1.060)	0.978 (0.899; 1.064)	0.965 (0.867; 1.073)[b]
**Leeds, Chronic (n=60)**	0.978 (0.926; 1.033)	0.941 (0.877; 1.008)	0.979 (0.919; 1.043)	0.959 (0.868; 1.060)	0.900 (0.773; 1.046)

[†] $p < .10$, * $p < .05$, ** $p < .01$

[a] NA=not applicable

[b] Three years in a row with at least intermittent colonization but not three years in a row with chronic colonization.

Effects (ratios, with 95% CI) of the presence of *A. fumigatus* ≥1 and ≥2 at a given year as well as the chronic and intermittent colonization by Leeds on ppFEV1, separately for five different time ranges.

Leeds criteria are for *P. aeruginosa* in CF patients and define chronic colonization as >50% positive sputum cultures and intermittent colonization as ≤50% positive sputum cultures in the last 12 months with a minimum of four cultures per patient and year

The effects are calculated within individuals and adjusted for the quadratic association with age.

A significant decrease in lung function with 4.5 % was detected the same year of acquisition only in CF patients with intermittent colonization by Leeds. However, CF patients chronically colonized by Leeds three years in a row did not associate with a

significant decrease in lung function, these associations were the strongest in absolute terms.

Table S2: Possible parameters that may predict the impact of *A. fumigatus* on lung function adopting different definitions.

Parameters	The year of detection	One year after detection	Two years after detection	Two years in a row	Three years in a row
***A. fumigatus* ≥2 at a given year**					
Age	0.999	0.999	0.999	NA	0.999
Gender	0.974	0.937	0.976	NA	1.031
Genotype 1 [a]	1.013	1.099	1.038	NA	1.11
Genotype 2[b]	1.008	1.028	1.018	NA	0.903
Pancreas insufficiency	1.08	1.115	1.159	NA	0.973
Diabetes 1[c]	1.034	0.925	1.014	NA	0.857
Diabetes 2[d]	1.167	1.437	1.258	NA	0.894
Number of iv[e] antibiotic treatments	0.963	0.925	0.953	NA	0.964
25 hydroxy vitamin D	1.001	**1.002***	1.001	NA	1.001
BMI (adults)	1.021	1.077	0.95	NA	0.903
ESR[f]	0.999	0.999	1	NA	1.005†
IgG	1.003	0.991	0.996	NA	1.005
Co-colonization with *C. dubliniensis*	0.982	1.008	1.092	NA	1.031
Co-colonization with *C. albicans*	1.03	1.015	1.065†	NA	1.043

Co-colonization with *P. aeruginosa*	**1.073***	1.000	1.011	NA	1.104
**Leeds, Intermittent**					
Age	0.999	0.999	0.996†	1	**0.99***
Gender	0.915	0.954	1.019	0.999	1.089
Genotype 1 [a]	1.13	1.052	1.094	1.157	1.202
Genotype 2[b]	1.06	0.851	0.962	0.909	0.814
Pancreas insufficiency	0.887	0.706	0.801	0.918	0.979
Diabetes 1[c]	0.994	0.977	0.994	0.851	**0.739***
Diabetes 2[d]	1.384	0.93	1.089	NA	NA
Number of iv[e] antibiotic treatments	1.147	1.086	1.104	1.271	1.192
25 hydroxy vitamin D	1.002	1.001	1.002	1.002	**1.004†**
BMI (adults)	0.888†	1.271	0.979	0.963	0.888
ESR[f]	1.003	0.996	0.995	1.006	1.005
IgG	1.016†	0.999	0.983	0.997	0.998
Co-colonization with *C. dubliniensis*	1.028	0.997	0.997	0.924	0.618
Co-colonization with *C. albicans*	1.055	1.076	1.134	1.056	1.114
Co-colonization with *P. aeruginosa*	1.059	0.921	1.040	0.971	**1.389***
**Leeds, Chronic**					
Age	1.001	1.001	0.997	0.998	0.994
Gender	1.048	1.041	1.039	1.205	1.628
Genotype 1 [a]	1.122	1.083	0.993	1.179	0.274

Genotype 2[b]	0.899	0.94	0.872	0.822	0.229
Pancreas insufficiency	1.023	1.044	0.94	1.127	NA
Diabetes 1[c]	0.927	0.933	0.908	0.968	0.526
Diabetes 2[d]	NA	NA	NA	NA	NA
Number of iv[e] antibiotic treatments	1.118	0.999	0.965	0.954	0.784
25 hydroxy vitamin D	1.002	1	1.002	1.005†	NA
BMI (adults)	1.129	**1.536***	NA	NA	NA
ESR[f]	1.001	1.004	1.005	1	0.965
IgG	1.005	0.986†	0.998	1	0.999
Co-colonization with *C. dubliniensis*	0.922	0.875	1.023	0.556	0.485
Co-colonization with *C. albicans*	1.064	1.088	1.104	**1.39****	**1.626****
Co-colonization with *P. aeruginosa*	**1.180****	**1.191****	**1.094**	**1.432†**	**1.794****

† $p < .10$, * $p < .05$, ** $p < .01$

[a] F508del homozygotes, [b] F508del heterozygotes, [c] Insulin treated diabetes mellitus, [d] Diet treated diabetes mellitus, [e] intravenous, [f] erythrocyte sedimentation rate.

Co-colonization with *P. aeruginosa, C. albicans*, higher BMI in adults, higher 25-hydroxy vitamin D and F5908del heterozygosity showed to be protective against the negative association of *A. fumigatus* colonization on lung function (OR >1). In contrary, increasing age and insulin treated CFRD were associated with a promotion of the predicted negative effect (OR<1)
